# Conserved Patterns of Microbial Immune Escape: Pathogenic Microbes of Diverse Origin Target the Human Terminal Complement Inhibitor Vitronectin via a Single Common Motif

**DOI:** 10.1371/journal.pone.0147709

**Published:** 2016-01-25

**Authors:** Teresia Hallström, Birendra Singh, Peter Kraiczy, Sven Hammerschmidt, Christine Skerka, Peter F. Zipfel, Kristian Riesbeck

**Affiliations:** 1 Department of Infection Biology, Leibniz Institute for Natural Product Research and Infection Biology, Hans Knöll Institute, Jena, Germany; 2 Clinical Microbiology, Department of Translational Medicine, Lund University, Malmö, Sweden; 3 Institute of Medical Microbiology and Infection Control, University Hospital of Frankfurt, Frankfurt, Germany; 4 Department Genetics of Microorganisms, Interfaculty Institute for Genetics and Functional Genomics, University of Greifswald, Greifswald, Germany; 5 Faculty of Biology, Friedrich Schiller University, Jena, Germany; University of Kentucky College of Medicine, UNITED STATES

## Abstract

Pathogenicity of many microbes relies on their capacity to resist innate immunity, and to survive and persist in an immunocompetent human host microbes have developed highly efficient and sophisticated complement evasion strategies. Here we show that different human pathogens including Gram-negative and Gram-positive bacteria, as well as the fungal pathogen *Candida albicans*, acquire the human terminal complement regulator vitronectin to their surface. By using truncated vitronectin fragments we found that all analyzed microbial pathogens (*n* = 13) bound human vitronectin via the same C-terminal heparin-binding domain (amino acids 352–374). This specific interaction leaves the terminal complement complex (TCC) regulatory region of vitronectin accessible, allowing inhibition of C5b-7 membrane insertion and C9 polymerization. Vitronectin complexed with the various microbes and corresponding proteins was thus functionally active and inhibited complement-mediated C5b-9 deposition. Taken together, diverse microbial pathogens expressing different structurally unrelated vitronectin-binding molecules interact with host vitronectin via the same conserved region to allow versatile control of the host innate immune response.

## Introduction

Upon infection and colonization of the human host, infectious agents are immediately attacked by the complement system, which forms the first and central line of the host innate immune defense. Activation of the complement system leads to generation of antimicrobial compounds, release of anaphylatoxins, C3b deposition on surfaces (opsonisation) and formation of the terminal complement complex (TCC) [1]. The complement system is activated by three major pathways; the alternative pathway is spontaneously activated on surfaces and foreign particles, and the classical and lectin pathways are induced by specific pattern recognition molecules and by antibody-antigen complexes [2]. All three pathways converge and form C3 convertases and this activation step is followed by formation of C5 convertases, release of potent inflammatory C5a and in the assembly of the TCC. The terminal pathway is initiated upon generation of C5b by the C5 convertase. Subsequently C6 and C7 bind to this newly formed C5b, and the C5b-7 complex attaches to and subsequently inserts into the target membrane. Further, binding of C8 and C9 results in formation of the C5b-9 complex (TCC) and in polymerization with C9 [3]. When C5b-9 is inserted into the target membrane the osmotic pressure changes and results in cell lysis. Complement activation can lead to extensive damage of host cells and tissues, and therefore a series of regulators protect the host surfaces [4].

Many, potentially even all, microbes including bacteria in the normal flora (*i*.*e*., the microbiome) resist and control the host complement attack at different levels. Despite the diversity of microbes, any pathogen faces the same type of host innate immune response due to pattern recognition, and in order to establish colonization and subsequent infection the pathogen must inactivate or overcome this central host defense system. Intriguingly, pathogens use related evasion strategies to control the alternative, lectin and classical pathway of complement activation as they bind the human complement regulators Factor H (FH), Factor H like protein 1 (FHL-1) and C4b binding protein (C4BP) as well as plasminogen to their surface [5]. Destructive complement effector functions including the release of potent inflammatory compounds and finally the formation of the TCC to resist complement-mediated killing are thus blocked by the attacking pathogens. Microbial pathogens also evade the destructive complement attack by expressing molecules mimicking complement regulators, producing proteases degrading complement components and inhibitors blocking various steps of the complement cascade [5–7].

In addition to utilization of the alternative and classical/lectin pathway regulators, many microbes acquire the human terminal pathway inhibitor vitronectin for immune escape [5,6,8]. Vitronectin can be found in plasma in two forms (75 and 65 kDa) at a concentration ranging from 200 to 700 μg/ml [8]. The glycoprotein binds C5b-7 at the metastable membrane-binding site and thereby inhibits membrane insertion of the complex, but can also directly inhibit C9 polymerization [9]. Vitronectin is composed of multiple domains including a somatomedin-B domain, the integrin receptor binding motif Arg-Gly-Asp (RGD), hemopexin-like domains, and three heparin-binding domains (HBD) [10,11]. In addition to its function as a soluble protein in plasma and at mucosal surfaces, vitronectin as a multimer is anchored to the extracellular matrix (ECM) and interacts with integrins promoting eukaryotic cell spreading, migration and adhesion [8,10,12]. When bacteria are coated with vitronectin they more efficiently adhere to host cells due to “bridging” with integrins, and this offensive strategy may also result in increased invasion [13–15].

To further characterize whether acquisition of human vitronectin is a common evasion strategy used by different pathogens independently of phylum we analysed vitronectin acquisition by a panel including 13 Gram-negative and Gram-positive bacteria, as well as *Candida albicans*. All microbial pathogens studied bound human vitronectin that was functionally active as monitored by inhibition of the TCC. Importantly, pathogens and their corresponding interacting proteins bound the same region comprising 23 amino acids in the C-terminal part of the vitronectin molecule. Our results reveal that acquisition of vitronectin is a general and highly conserved feature that infectious agents of diverse origins have successfully adopted.

## Material and Methods

### Bacterial Strains and Culture Conditions

Bacterial and fungal strains (Table 1) were routinely cultured on appropriate agar or in liquid broth at 37°C. Transformed *Escherichia coli* M15 or BL21 expressing Lpd, Msf or Efb were grown in Luria Bertani (LB) broth supplemented with 100 μg/ml carbenicillin and/or 25–50 μg/ml kanamycin.

**Table 1:** Microbial strains used in the present study.

Species	Strain	Characterization	Reference
**Gram-negative bacteria**			
*Acinetobacter caleoaceticus*	ATCC 23055		
	KR824		This study
*A*. *baumannii*	ATCC 19606		
	KR810	Urine isolate	[48]
*Bordetella pertussis*	CCUG 33528		
*Haemophilus influenzae* type b (Hib)	RM804		[15]
	Minn A		This study
	ATCC 49247		
	CCUG 18095		
Non-typeable *H*. *influenzae* (NTHi)	NTHi 3655		[16]
*Helicobacter pylori*	CCUG 17875		
	KR697	Antrum Biopsy (gastritis sample)	This study
*Legionella pneumophila*	CCUG 33152		
*Moraxella catarrhalis*	Bc5		[49]
	RH4		[50]
	O35E		
*Neisseria meningitidis*	CCUG 3269		
	FAM20		[51]
	KR822	Likvor isolate	This study
*Pseudomonas aeruginosa*	SG137		[52]
	NCTC 10662		[52]
	ATCC 27853		[52]
**Gram-positive bacteria**			
*Staphylococcus aureus*	ATCC 25923		
	ATCC 29213		
	ATCC BAA-1026		
*Streptococcus pneumoniae*,	ATCC 25770		
	ATCC 49619		
	D39		[53]
	KR823	Blood isolate	This study
*Streptococcus pyogenes*	CCUG 25770		
	CCUG 25570		
	NCTC 9994		
**Fungi**			
*Candida albicans*	CCUG 19915		
	CCUG 32723		
	CA1000		[54]

#### Expression of recombinant proteins

Recombinant fragments of human vitronectin (aa 80–396, 80–379, 80–373, 80–363, 80–353, 80–339, 80–330, 80–320 and 80–229) and vitronectin deletion mutants (Δ352–362, Δ362–374 and Δ352–374) were expressed as previously described [16,17]. Briefly, HEK293T cells were grown in three triple flasks (Nunc, Roskilde, Denmark) to 80% confluence using advanced DMEM supplemented with penicillin (100 U/ μl) and streptomycin (100 μg/ml) and 1% FCS in 37°C with 5% CO_2_. Transfected cells were incubated for 3 days at 37°C with 5% CO_2_ followed by harvest of supernatants [18]. A similar volume of advanced DMEM was once again added to the cells and the procedure was repeated after 3 days. Vitronectin having a His-tag was secreted into the medium and was purified by Ni-NTA chromatography.

Recombinant *H*. *influenzae* Hsf (*Haemophilus* surface fibrils), *H*. *influenzae* PE (protein E), *M*. *catarrhalis* UspA2H (ubiquitous surface protein A2), *P*. *aeruginosa* Lpd (Lipoamide dehydrogenase) [19], *S*. *pneumoniae* PspC (pneumococcal surface protein C), *S*. *pyogenes* Scl (streptococcal collagen like protein) and *C*. *albicans* Gpm1 (phosphoglycerate mutase 1) were expressed and purified as previously described [15,17,20–23]. *N*. *meningitidis* Msf (meningococcal surface fibril) (aa 52–473) was cloned in the *E*. *coli* pET200/D-TOPO vector expression system (Invitrogen, Grand Island, NY) using the primers Msf 5´-CACCACCGATACCGATGAAGATGAAGAG-3 and Msf 5´-TCAGGCATCCTTGCTG CCGACATTC-3´ [24]. Protein expression was induced by 1 mM isopropyl β-D-thiogalactoside. All recombinant proteins were purified by Ni-NTA affinity chromatography using HisTrap chelating columns and an Äkta FPLC system (GE Healthcare, Freiburg, Germany).

### Protein Labeling and Direct Binding Assays

Vitronectin was purified from human plasma as previously described [25]. Purified plasma vitronectin and recombinant vitronectin^80-396^ were labeled with iodine (0.05 M; Perkin Elmer, MA) per mole of protein using the chloramine T method [26]. The specific activity of [^125^I]-labeled vitronectin and vitronectin^80-396^ were ≈1.25 x 10^7^ cpm/ μg. The different microbial strains were grown overnight on agar plates and washed in PBS supplemented with 1% BSA. Bacteria (2x10^8^) or *C*. *albicans* (10^6^) were incubated with [^125^I]-labeled vitronectin or vitronectin^80-396^ for 1 h at 37°C. After incubation, bacteria were extensively washed and binding of iodine-labeled proteins to bacterial cells was measured in a liquid scintillation counter (Tricarb Scintillation counter; Perkin Elmer, Upplands Väsby, Sweden).

#### ELISA

Microtiter plates (F96 Polysorb, Nunc-Immuno Module) were coated with intact bacteria (10^8^/well), microbial proteins (5 μg/ml) or BSA (5 μg/ml) overnight at 4°C. Plates were washed four times with PBS containing 0.1% Tween 20 (PBS-T) and blocked for 1 h at room temperature (RT) with PBS supplemented with 2% BSA (PBS-BSA). After washings, the plates were incubated for 1 h at RT with vitronectin (5 μg/ml) or equimolar amounts of the various recombinant vitronectin fragments (70 nM). Thereafter, wells were washed and incubated with a rabbit anti-human vitronectin pAb (1:1000), followed by horseradish peroxidase (HRP)-conjugated swine anti-rabbit pAbs (1:2500). The reaction was developed with 1,2-phenylenediamine dihydrochloride (OPD, DakoCytomation, Glostrup, Denmark) or 20 mM tetramethyl-benzadine with 0.1 M potassium citrate and the absorbance was measured at 492 nm or 450 nm. In the competition assay, the microbial proteins were incubated with vitronectin (5 μg/ml) that was pre-incubated with heparin (10 μM) or NaCl (1 M).

### Binding Assay with Intact Microbes

The binding of vitronectin fragments to microbes was assayed in the fluid phase. Bacteria (10^6^) were incubated with the various vitronectin fragments (0.2 μM) in PBS for 1 h at 37°C. Unbound proteins were removed by three washes with PBS followed by centrifugation at 10000g. Thereafter, bacterial pellets were incubated with 1xSDS-PAGE (containing β-mercaptoethanol) loading buffer for 10 min at 95°C and centrifuged for 5 min at 20000×g. Supernatants (10–15 μl) were subjected to SDS-PAGE, transferred to a membrane and analyzed by Western blotting using a goat anti-vitronectin pAb (AbD Serotec, Kidlington, Oxford, UK) (1:2000) for 1 h followed by HRP-conjugated donkey anti-sheep pAb (AbD Serotec) (1:4000) for 40 min. Development was performed with an ECL Western blotting kit (Pierce, Thermo Scientific, Rockford, IL).

### Flow Cytometry Analysis

To analyze whether vitronectin bound to the microbial surface is functionally active, the microbial pathogens were incubated with vitronectin purified from human plasma (10–50 μg/ml) (Corning, Kaiserslautern, Germany) for 30 min at 37°C. After three washes, microbes were incubated with C5b-6 (1 μg/ml) and C7 (1 μg/ml) for 5 min, and thereafter C8 (0.4 μg/ml) and C9 (1 μg/ml) were added for 30 min at 37°C. All these complement factors were purchased from Complement Technology (Tyler, TE). After washing, deposited C5b-9 was detected by mouse anti-human C5b-9 mAb (Abcam, Cambridge, UK) followed by Alexa fluor-647-conjugated polyclonal goat anti-mouse pAb (Abcam). After two additional washes, microbes were analyzed by flow cytometry (EPICS XL-MCL; Coulter, Hialeah, FL). All incubations were kept in a final volume of 100 μl PBS-BSA and washings were done with the same buffer. Primary and secondary pAb were added separately as negative controls for each strain analyzed.

### TCC Deposition Assay

Microtiter plates (F96 Medisorb, Nunc-Immuno Module) were coated with microbial proteins (5 μg/ml) overnight at 4°C. The plates were washed four times with PBS-T and blocked for 1 h at RT with PBS-BSA. After washings, the plates were incubated for 1 h at RT with vitronectin (50 μg/ml). After incubation, the wells were washed and incubated with C5b-6 (1.5 μg/ml) and C7 (1 μg/ml) for 10 min at RT, and thereafter C8 (0.2 μg/ml) and C9 (1 μg/ml) were added and incubated for 30 min at 37°C. C5b-9 deposition was detected with mouse anti-human C5b-9 mAb (1:1000) (Abcam) and HRP-conjugated swine anti-mouse pAb (1:2500) (DakoCytomation). The reaction was developed with 1,2-phenylenediamine dihydrochloride (OPD; DakoCytomation) and the absorbance was measured at 492 nm.

### Statistics

Results were assessed by Student´s *t*-test for paired data. *p* ≤ 0.05 was considered to be statistically significant. *, *p* ≤ 0.05; **, *p* ≤ 0.01; ***, *p* ≤ 0.001.

## Results

### An Array of Pathogens Interact with Vitronectin

**Fig 1:**
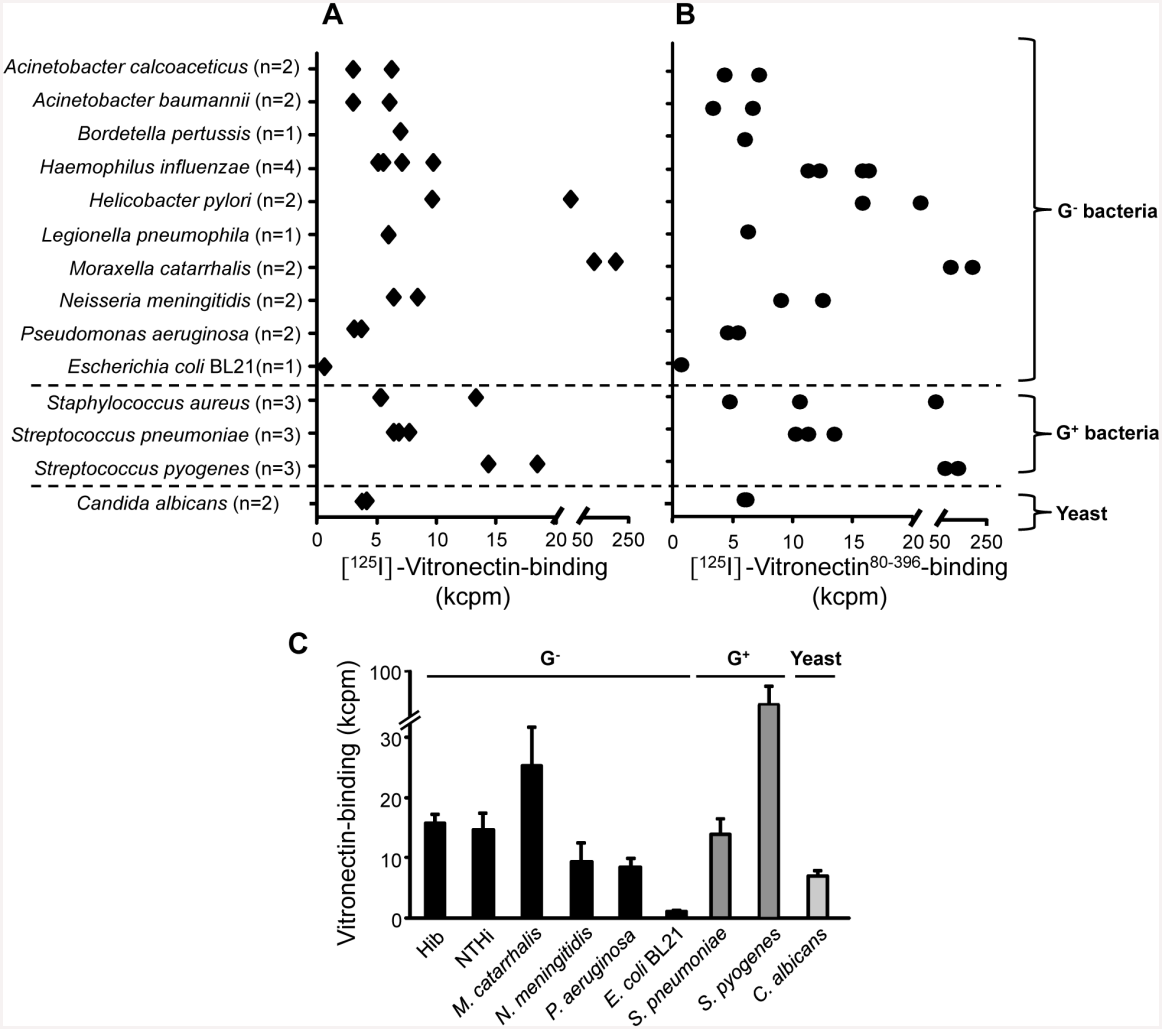
Pathogenic microbes bind the terminal complement pathway inhibitor vitronectin. A series of Gram-negative and Gram-positive bacterial species and *Candida albicans* were grown overnight and incubated with [^125^I]-labeled vitronectin purified from plasma (A) or recombinant vitronectin^80-396^ (B). After washing, bound vitronectin was determined by liquid scintillation counting. The mean values of three experiments are shown. In C, the relative binding of [^125^I]-labeled vitronectin to selected microbes are shown. The mean values of three experiments are shown with error bars indicating standard deviations (SD).

To define whether acquisition of human vitronectin is a common feature of pathogens and compare individual differences, we comprehensively analyzed vitronectin binding to several microbes including Gram-negative (*n* = 9) and Gram-positive (*n* = 3) bacterial species in addition to the human pathogenic yeast *C*. *albicans*. Microbes were incubated with iodine-labelled vitronectin purified from human plasma and recombinant vitronectin (amino acids 80–396), followed by washing and detection of bound vitronectin by scintillation counting. Importantly, all microbes bound vitronectin, however, different isolates varied in their capacity to bind this complement regulator (Fig 1A). Binding was confirmed with recombinant vitronectin^80-396^ (Fig 1B). Seven different bacterial species in addition to *C*. *albicans* were selected for further characterization in downstream experiments (Fig 1C).

### Human Pathogens Bind Vitronectin via the C-Terminal Region

**Fig 2:**
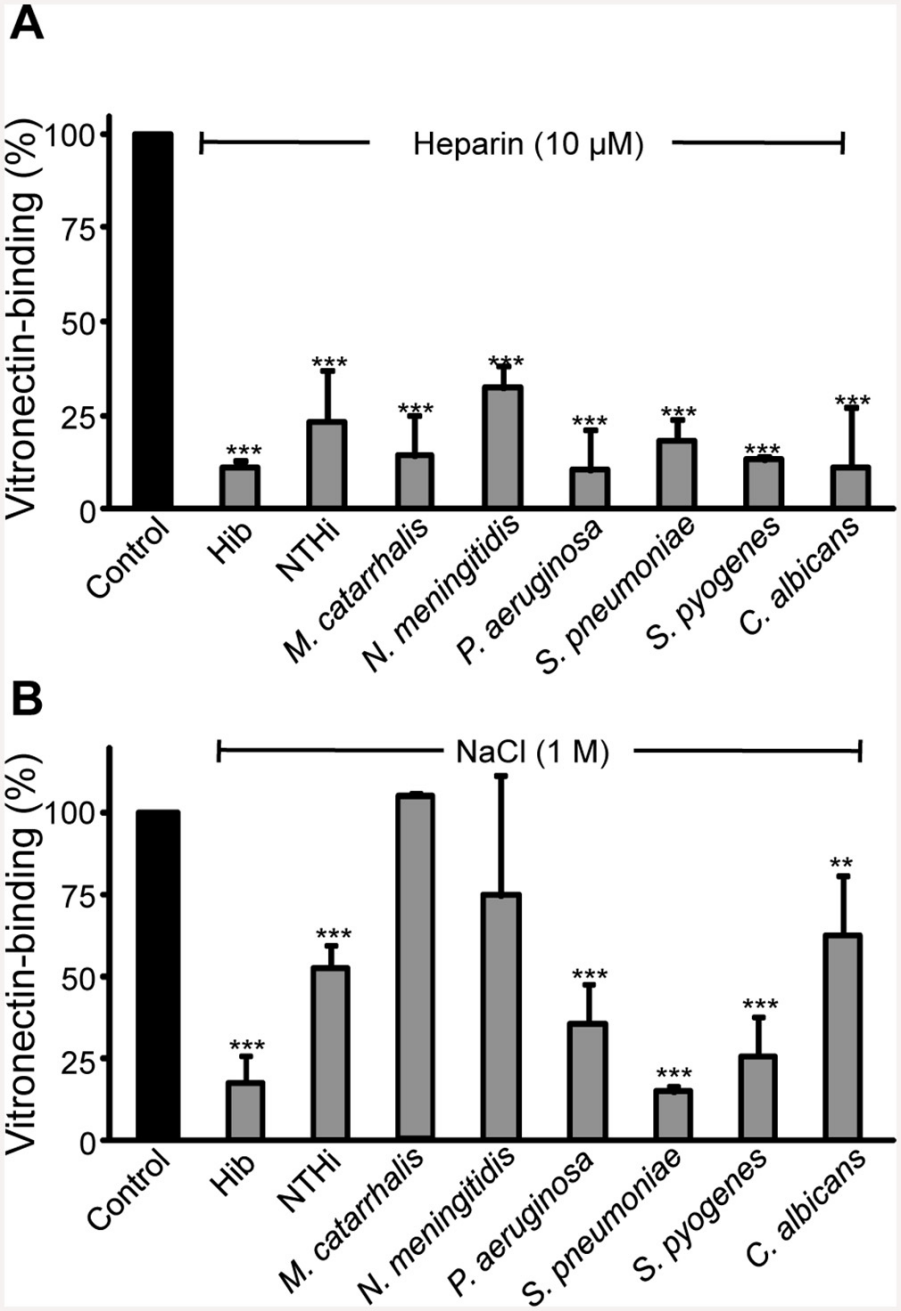
The binding of vitronectin is inhibited by heparin and high ionic strength. Heparin and NaCl inhibited the binding of [^125^I]-labeled vitronectin to the microbial pathogens. The microbial pathogens were incubated with [^125^I]-labeled vitronectin and 10 μM heparin (A) or 1 M NaCl (B), followed by washing and determination of radioactivity associated with the pellet. The vitronectin binding of each microbe in the absence of competitor was defined as 100%. The mean values of three experiments are shown with error bars indicating SD. Statistical significance of differences was estimated using Student’s *t* test. **, *p*≤ 0.01; ***, *p*≤ 0.001.

Vitronectin harbors several functional regions including three HBDs [10,11]. To determine whether pathogens bind vitronectin via HBDs, we first analyzed if heparin would influence binding of vitronectin to the microbial surface. Heparin (10 μM) significantly inhibited vitronectin binding by >75% to all species tested (Fig 2A). Assuming that ionic strength might influence binding of vitronectin to the selected microbes, the effect of NaCl was evaluated. As shown in Fig 2B, salt inhibited vitronectin binding to the majority of microbes and the binding was inhibited 25–85% by NaCl. NaCl did not, however, influence vitronectin binding to *M*. *catarrhalis*. In summary, binding of vitronectin occurred via HBDs, and the interaction was of ionic nature for most of the microbes.

**Fig 3:**
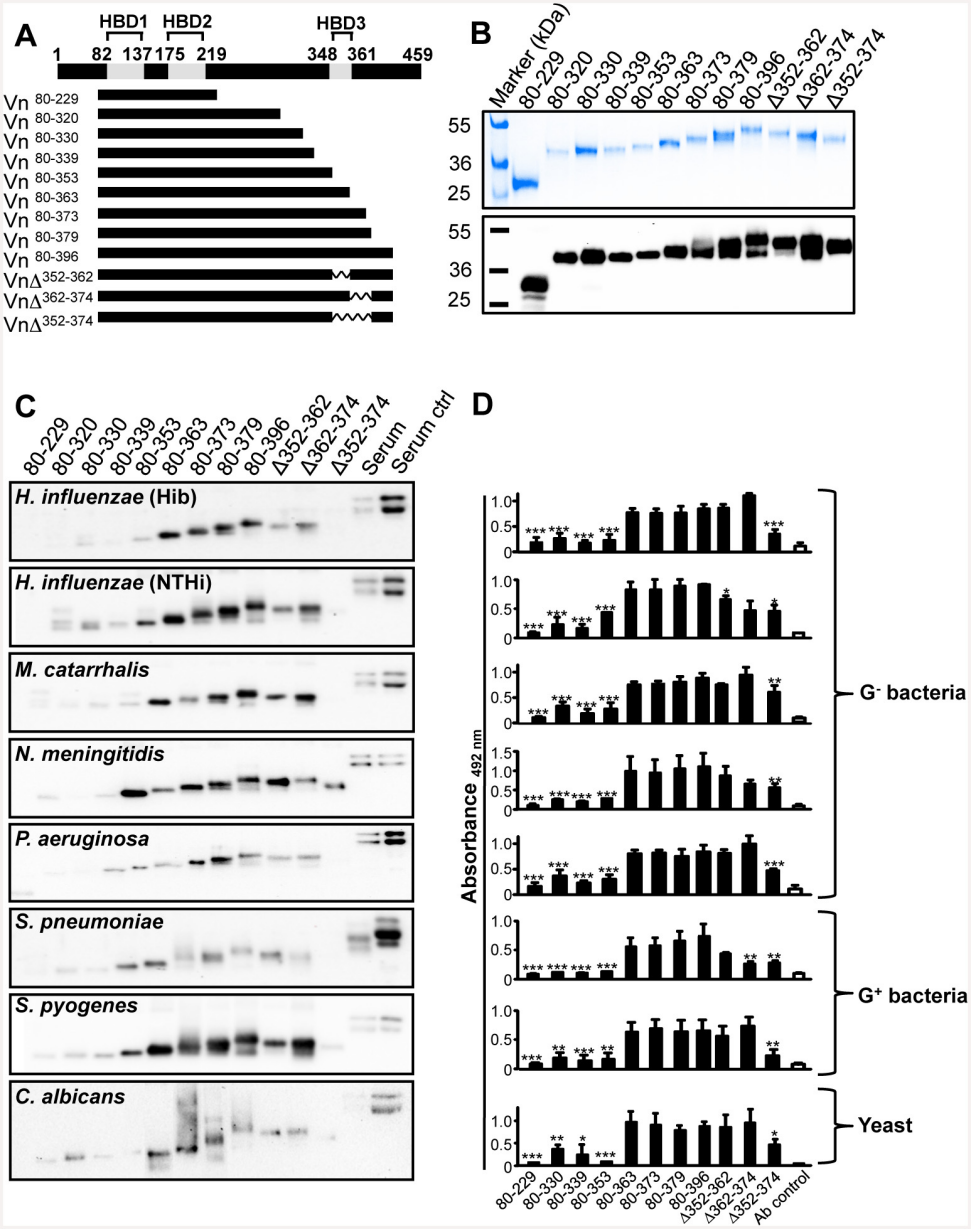
The microbial binding site is located within the third HBD. (A) Schematic representation of the different truncated vitronectin (Vn) fragments and deletion mutants used for analysis of vitronectin binding. (B) Eight different microbes, including Gram-negative and Gram-positive bacteria and *C*. *albicans* were incubated with truncated recombinant vitronectin fragments and three vitronectin deletion mutants. NHS was run in parallel as a positive control. Microbes were washed and total proteins were separated by SDS-PAGE gels, transferred to a membrane and bound vitronectin fragments were detected using an anti- vitronectin polyclonal antiserum. One representative experiment of three independent ones performed is shown. (C) Microbes were immobilized on microtiter plates and incubated with vitronectin fragments including deletion mutants. Bound fragments were detected with rabbit anti-vitronectin pAb and secondary HRP-conjugated goat anti-rabbit pAb. The mean values of three experiments are shown with error bars indicating SD. Statistical significance of differences was estimated using Student’s *t* test. *, *p*≤ 0.05; **, *p*≤ 0.01; ***, *p*≤ 0.001.

To localize the specific region(s) within the vitronectin molecule that was targeted by the various pathogens, a series of vitronectin deletion fragments (Fig 3A and 3B) were produced in a mammalian expression system and further tested for binding [16,17,19]. The pathogens were incubated with equimolar concentrations of vitronectin deletion variants followed by washing and separation of the whole cell proteome in SDS-PAGE. Thereafter, bound vitronectin fragments were identified by Western blotting. This approach revealed a relatively similar pattern for all the microbes analyzed, that is, all of them bound vitronectin^80-396^, vitronectin^80-379^ and vitronectin^80-363^. In contrast, the truncated fragments vitronectin^80-353^ vitronectin^80-339^, vitronectin^80-330^ and vitronectin^80-229^ bound with reduced or significantly lower intensity (Fig 3C). Our result thus suggested that 23 residues spanning from position 352 to 374 that include the HBD3 are important for binding. Despite some pathogens interacted with the fragments vitronectin^80-353^ and vitronectin^80-339^ when analyzed by Western blots, the quantitative direct binding assay revealed a clearly decreased binding of those to truncated vitronectin fragments when compared to the full length vitronectin molecule (amino acids 80–396) (Fig 3D).

### Microbial Proteins Acquire Vitronectin at the Same 23 Residues (Region 352–374)

**Fig 4:**
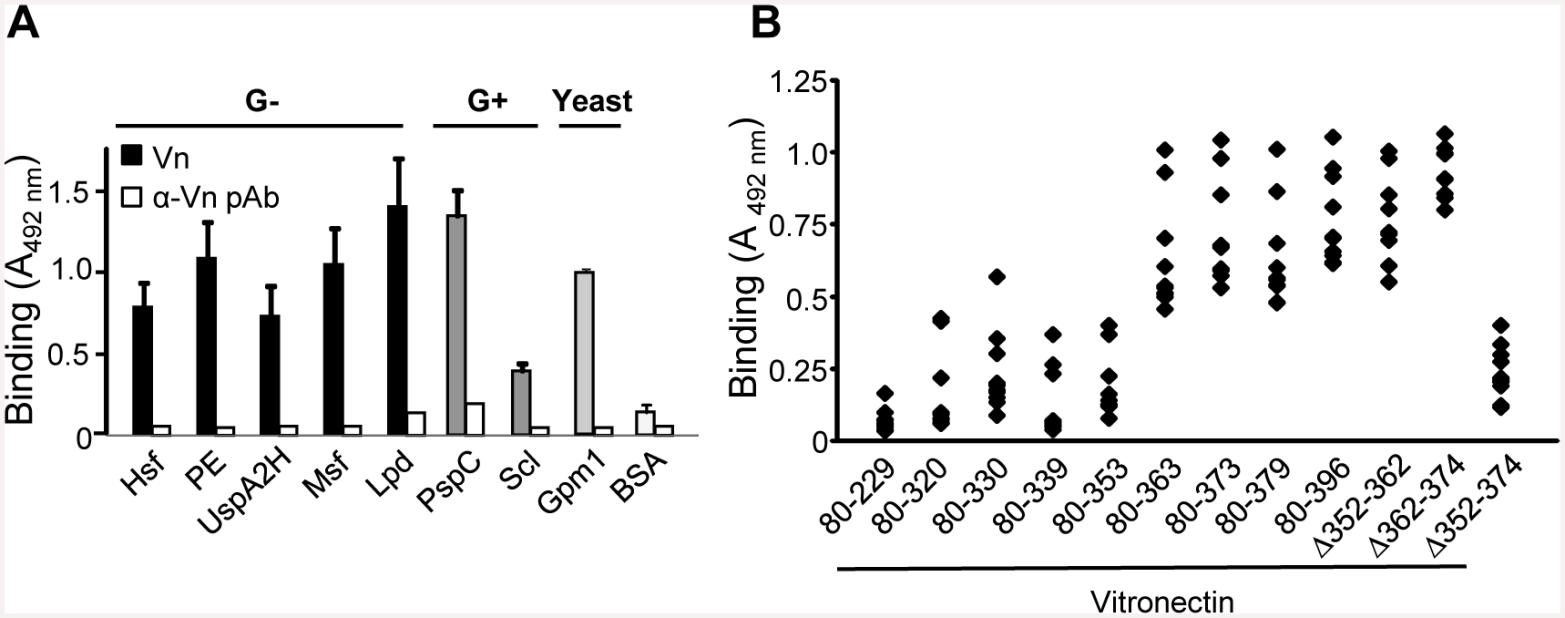
All microbial proteins use a common binding site within the vitronectin molecule. (A) Recombinant Hsf (Hib), PE (NTHi), UspA2H (*M*. *catarrhalis*), Msf (*N*. *menigitidis*), Lpd (*P*. *aeruginosa*), PspC (*S*. *pneumoniae*), Scl1 (*S*. *pyogenes*) and Gpm1 (*C*. *albicans*) (5 μg/ml) were immobilized in microtiter plates and incubated with vitronectin (5 μg/ml). (B) Microbial proteins were immobilized on microtiter plates and incubated with recombinant vitronectin fragments and deletion mutants. The diamonds represent the microbial proteins tested. In (A) and (B), bound vitronectin fragments and deletion mutants were detected with a vitronectin reacting polyclonal antiserum and HRP-conjugated anti-rabbit pAb. The mean values of three experiments are shown with error bars indicating SD. Binding to vitronectin was statistically significant (*p*≤ 0.001) for all microbial proteins.

Vitronectin-binding surface proteins have recently been identified for several of the selected pathogens (Table 2) and include Hsf (*H*. *influenzae type b*; Hib), PE (non-typeable *H*. *influenzae*; NTHi), UspA2H (*M*. *catharrhalis*), Msf (*Neisseria meningitidis*), Lpd (*P*. *aeruginosa*), PspC (*S*. *pneumoniae*), Scl (*S*. *pyogenes*) and Gpm1 (*Candida albicans*) [15–17,19,21,23,24]. We recombinantly expressed vitronectin-binding proteins either in *E*. *coli* or *Pichia pastoris* followed by Ni-NTA affinity chromatography. Binding of plasma vitronectin to the immobilized microbial proteins was determined by ELISA (Fig 4A). *Pseudomonas* Lpd and pneumococcal PspC bound vitronectin, whereas *Streptococcal* Scl showed a lower binding. In addition, we confirmed that HBDs are important for the interactions by adding heparin (10 μM) that resulted in an inhibition of the protein-protein interactions with 68–89% (data not shown).

**Table 2:** Pathogens and their known vitronectin-binding characteristics.

Pathogen	Microbial protein	HBD involved	Binding region within Vn (aa)	Reference
**Gram-negative bacteria**				
*Escherichia coli*		Yes		[44]
*Haemophilus influenzae* type b	Hsf	Yes	352–374	[15,55]
*Haemophilus ducreyi*	DsrA	N/A ^1^		[56,57]
*Helicobacter pylori*		N/A		[58]
*Leptospira interrogans*	LcpA	Yes		[59]
NTHi	PE	Yes	353–363	[16,20]
	PF	Yes	352–374	[29]
*Moraxella catarrhalis*	UspA2/A2H	Yes	312–396	[17,34,49]
*Neisseria gonorrhoeae*	Opa_50_	N/A		[45,60]
	OpaA	Yes		[61]
	Opc	N/A		[62]
		N/A		[63]
*Pseudomonas aeruginosa*	-	N/A		[13]
	Lpd	Yes	353–363	(submitted)
*Pseudomonas fluorescens*		N/A		[64]
*Rickettsia conorii*	Adr1	N/A		[65]
**Gram-positive bacteria**				
*Enterococcus faecalis*		N/A		[42]
*Staphylococcus aureus*	-	Yes		[40,44,66]
	Autolysin	N/A		[67]
*Staphylococcus epidermidis*	Autolysin	N/A		[39]
	Autolysin/Adhesin	N/A		[68]
	(Aae)			
*Streptococcus pneumoniae*	PspC	Yes	353–363	[21]
	Hic	Yes	353–363	[31]
	-	Yes		[14]
*Streptococcus pyogenes*		No	138–160	[30,44]
**Fungi**				
*Candida albicans*	Gpm1	Yes		[23]
	30 kDa protein	Yes		[69]
*Candida dubliniensis*		N/A		[70]
*Pneumocystis carinii*		Yes		[46]

^1^ N/A: not applicable

The majority of known microbial vitronectin-binding proteins recruit the complement regulator vitronectin via the C-terminal region [8]. To fully define whether this pattern was valid for 8 selected vitronectin receptors, *i*.*e*., Hsf, PE, UspA2H, Msf, Lpd, PspC, Scl1 and Gpm1, a series of vitronectin deletion mutants was included in our analysis. This approach revealed a highly conserved binding pattern and further confirmed that vitronectin fragments lacking the third HBD bound with much lower intensity (Fig 4B). Importantly, all purified proteins tested had a similar vitronectin-binding pattern as observed with the corresponding whole bacteria (Fig 3C). A clear pattern was thus observed, that is, despite various structures and phyla all microbial vitronectin-binding proteins target the same C-terminal amino acids spanning vitronectin 352 to 374.

### Vitronectin Bound to the Microbial Proteins Is Functionally Active and Inhibits TCC Formation

**Fig 5:**
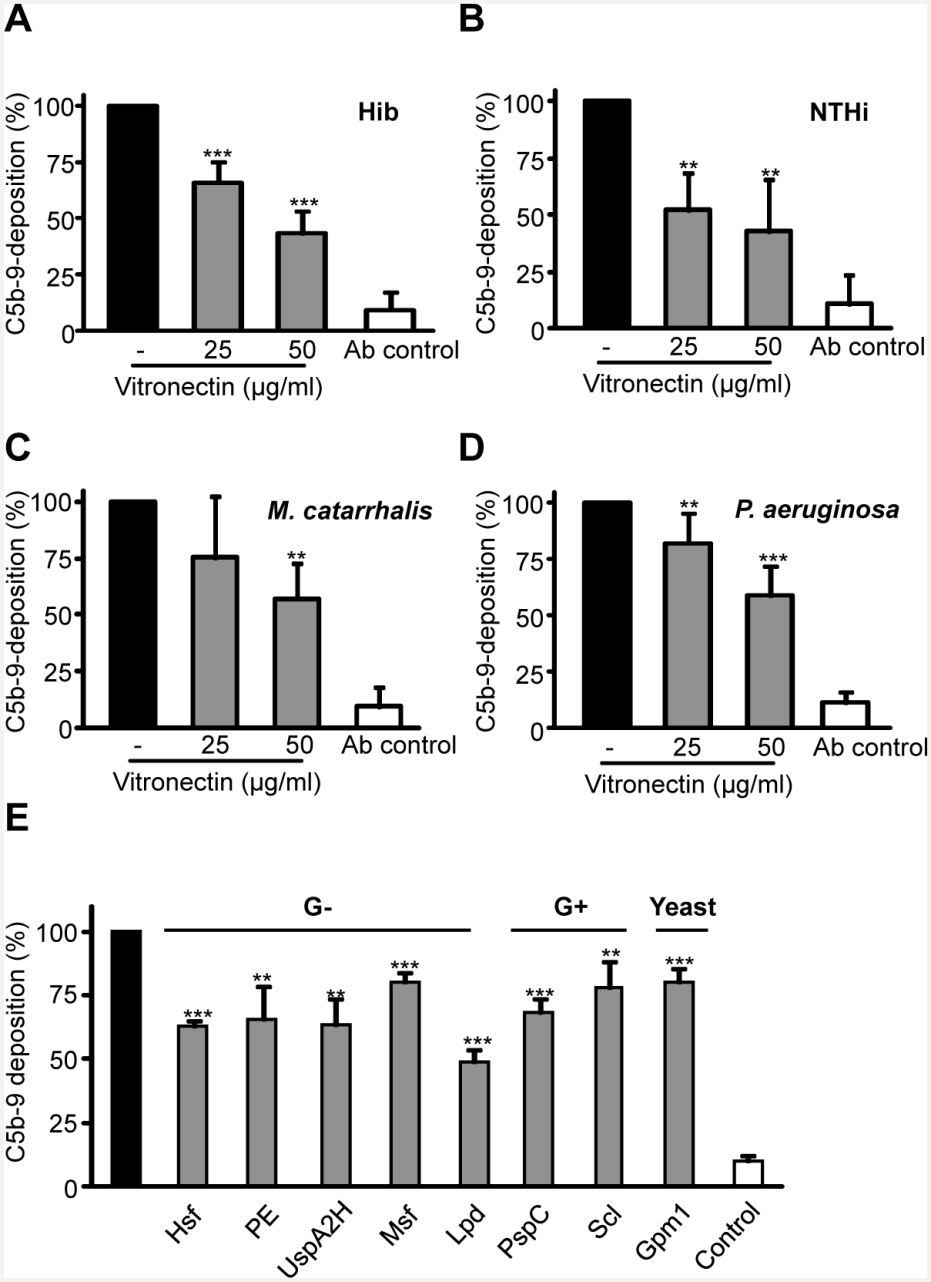
Vitronectin bound to intact bacteria inhibits C5b-9 deposition. Vitronectin (25–50 μg/ml) was bound to Hib (*A*), NTHi (*B*), *M*. *catarrhalis* (*C*) or *P*. *aeruginosa* (*D*), and after extensive washing C5b-6 and C7 were added. After incubation for 10 min, C8 and C9 were added, and thereafter C5b-9 deposition on the microbial surface was detected with a mouse anti-C5b-9 mAb and Alexa 647-conjugated anti-mouse pAb by flow cytometry. E, vitronectin (50 μg/ml) was bound to immobilized proteins, and after extensive washing C5b-6 and C7 were added. After 10 min incubation, C8 and C9 were added, and C5b-9 deposition was detected with a mouse anti-C5b-9 mAb and an HRP-conjugated anti-mouse polyclonal antiserum. The mean values from three independent experiments are shown with error bars indicating SD. **, *p* ≤ 0.01; ***, *p* ≤ 0.001.

To demonstrate that vitronectin bound to the microbial surface maintains the regulatory function and inhibits the terminal complement pathway, we analyzed whether vitronectin attached to the surface of four pathogens inhibits TCC deposition. To this end, vitronectin was bound to the surface of encapsulated (Hib) or non-typeable (NTHi) *H*. *influenzae*, *M*. *catarrhalis* or *P*. *aeruginosa*, and after washing, C5b-6 and C7 were supplemented followed by addition of C8 and C9. Thereafter, cell surface deposition of C5b-9 was determined by flow cytometry. When vitronectin was bound to all four Gram-negative bacteria, C5b-9 surface deposition was significantly inhibited and the effect was dose-dependent (Fig 5A–5D). Vitronectin at the highest concentration used (50 μg/ml) inhibited C5b-9 deposition by 57% (Hib), 57% (NTHi), 39% (*M*. *catharrhalis*) and 41% (*P*. *aeruginosa*). Thus, vitronectin inhibits C5b-9 deposition when bound to the bacterial cell surface.

In addition, we analyzed whether vitronectin maintained its regulatory function upon interaction with the microbial ligands. Vitronectin was allowed to bind to immobilized proteins, and after washing, C5b-6 and C7 were supplemented followed by addition of C8 and C9. After incubation, C5b-9 deposition was analyzed by a specific anti-human C5b-9 mAb. An inhibitory effect was observed, albeit C5b-9 deposition was decreased to a varying extent (Fig 5E). In conclusion, vitronectin attached to the surface of pathogens, or bound to the corresponding vitronectin ligands is functionally active and inhibits assembly of the terminal complement pathway and consequently TCC formation.

## Discussion

Microbial pathogens acquire host complement regulators by a range of diverse molecules localized on the outer surface. During the course of an infection, pathogens do not only rely on a single host protein for their survival and virulence, rather it is a multifunctional process. For example, adherence to host tissues is commenced by anchoring to several ECM proteins at the same time. Likewise, the resistance against complement is achieved by simultaneous recruitment of various host complement regulators, such as C4BP, Factor H, vitronectin or clusterin. This multiple approach leads to significantly enhanced survival of pathogens. The present study shows that 13 different pathogens bind the human complement regulator vitronectin, which inhibits the terminal pathway by blocking the membrane binding site of C5b-7 and polymerization of C9. Moreover, we extend previous findings by mapping the binding site(s) of pathogen ligands on the vitronectin molecule [9,27]. This common complement evasion strategy apparently is conserved for Gram-negative and Gram-positive bacteria as well as for the fungus *C*. *albicans*.

To deceive the host, microbial surface proteins of a particular species are highly diverse in structure, and furthermore display large variations between various microbes promoting a variety of functions. Some of the surface molecules are specifically known for a single function, whereas others simultaneously bind multiple complement regulators [5,6]. Microbes acquire host complement regulators very precisely by keeping their original function intact. This function is preserved by recognizing specific regions of those regulator molecules while leaving other host-utility functional regions unoccupied. Interestingly, it has been observed that bacterial pathogens from different phyla recognize a similar region (complement control protein; CCP 20) of human Factor H [28]. Similarly, CCP 1–3 domains of C4BP are recognized by several bacterial and fungal pathogens [6].

Vitronectin consists of a multidomain structure, the N-terminal SMB domain is responsible for regulation of the host fibrinolytic system and RGD recognizes integrins [8,10,12]. In addition to these functions, vitronectin has also binding sites for collagen, plasminogen, complement components, heme, and heparin [17,29]. Liang and co-workers described for the first time that hemopexin-type repeats of vitronectin are recognized by *S*. *pyogenes* [30]. Later on heparin-binding sites were mapped in those hemopexin repeats [11]. Since the discovery of HBDs in the vitronectin molecule, several pathogens including *E*. *coli*, *C*. *albicans*, *H*. *influenzae*, *M*. *catarrhalis*, *Neisseria gonorrhoeae*, *S*. *pneumoniae* and *P*. *aeruginosa* have been analyzed for their interactions with vitronectin (Table 2). Among three distinguished HBDs, HBD-3 was later recognized as a prime motif recognized by some pathogens [15,16,29,31]. Moreover, in addition to HBD-3, *N*. *meningitidis* also recognizes the N-terminal region of the vitronectin molecule [32]. In our study, various truncated fragments of HBD-3 were used for fine mapping the binding domain of an array of selected pathogens in addition to a few known vitronectin-binding proteins for verification of specific protein-protein interactions. Our results suggest that vitronectin-binding proteins selected from different pathogens have a common and conserved binding motif on the vitronectin molecule. Surprisingly, salt affected the majority of vitronectin-microbe interactions but to a various extent, suggesting that the protein-protein interactions may involve ionic interactions for some pathogens, but not for all (Fig 2B). Most importantly and also intriguingly, despite their diverse origin and a lack of sequence homology, the microbial proteins bound the same region of the human vitronectin molecule (aa 352–374).

Vitronectin inhibits both the assembly and formation of TCC by inhibiting the C5b-7 complex at its membrane-binding site and the C9 polymerization (8, 10). The insertion of the complex into the cell membrane is thereby inhibited, preventing lysis of the microbe in question. When bound to a microbial protein, the functional part of vitronectin, *i*.*e*., aa 51–310 is exposed to the outside and thus is able to inhibit the terminal pathway by blocking C5b-9 deposition [33]. It is of outmost importance for the microbe to have a functional vitronectin domain available in order to survive the direct attack of the terminal pathway or block further TCC-mediated functions. The inhibition of the terminal pathway and its bactericidal activity is particularly important for Gram-negative bacteria such as *H*. *influenzae*, *M*. *catarrhalis*, and *P*. *aeruginosa* of which all are more or less sensitive to TCC-mediated killing. All three bacterial species are more rapidly killed in vitronectin-depleted serum than in normal human serum suggesting that bound vitronectin protects against complement attack [15,19,20,34]. Vitronectin is hence functionally active when bound to the surface of these bacteria and inhibits TCC deposition. In contrast, TCC is not considered relevant for killing of Gram-positive bacteria and human pathogenic fungi. Due to their thick peptidoglycan layer and complex cell wall, Gram-positive bacteria and pathogenic fungi are resistant to TCC-mediated pore formation and lysis. Recent data suggest, however, that TCC deposition may also occur on the surface of Gram-positive bacteria, including amongst others *S*. *pyogenes* and *S*. *pneumoniae* [35]. In addition to its function in the complement system, TCC has also been shown to be involved in pro-inflammatory reactions and to amplify the inflammatory response by regulating cell functions. TCC modulates P-selectin expression, increases the synthesis of inflammatory mediators such as leukotrienes and prostaglandins and increases the synthesis of oxygen-derived free radicals and secretion of IL-8 and MCP-1 from HUVECs [33–37]. Furthermore, TCC acts synergistically with TNF-α to increase the ICAM-1 and E-selectin expression, and is involved in inflammasome activation [36–38]. Thus, inhibition of the TCC-mediated actions by utilizing vitronectin is an efficient strategy to avoid complement-mediated killing of Gram-negative bacteria in addition to induction of the pro-inflammatory response that is important for all pathogens including Gram-positive bacteria and fungi.

Binding of the microbial pathogens to exposed ECM components such as vitronectin may contribute to bacterial adherence, which is an essential step in the microbial pathogenesis. One hypothesis is that these interactions contribute to the spread of bacteria through tissue barriers into secondary infection sites. Pathogens use vitronectin for ECM binding and for adhesion to human cells, as shown for *H*. *influenzae*, *S*. *pneumoniae*, *S*. *pyogenes*, *S*. *epidermidis*, *S*. *aureus* and *Enterococcus faecalis* [14,15,39–44]. Vitronectin also mediates attachment of *N*. *gonorrhoeae*, *C*. *albicans* and *Pneumocystis carinii* to human cells, including endothelial and epithelial cells [23,45–47].

In conclusion, we have identified a common binding site on the multifunctional vitronectin molecule that is recognized by several pathogens. Binding via this specific region allowed microbes to utilize the complement inhibitory function of vitronectin, as the N-terminal functional region of vitronectin was not shielded. Thus, the ability of pathogenic microbes to bind vitronectin contributes to evasion of direct lysis by complement, which is an important step in pathogenesis. Our expanding knowledge of how microbes interact with the complement system is of outmost importance, not only to increase the understanding of the infection process, but also for the development of new vaccines and alternative therapeutic options.
